# Mucinous cystic neoplasm of the liver with extrahepatic growth presenting with ascending cholangitis diagnosed by endoscopic ultrasound features: a case report

**DOI:** 10.1186/s13256-017-1560-4

**Published:** 2018-02-15

**Authors:** Tanawat Pattarapuntakul, Bancha Ovartlarnporn, Jaksin Sottisuporn

**Affiliations:** NKC Institute of Gastroenterology and Hepatology, Faculty of Medicine, Songklanagarind Hospital, Prince of Songkla University, Hatyai, 90110 Songkhla Thailand

**Keywords:** Mucinous cystic neoplasm of the liver with extrahepatic growth, Biliary cystadenoma, Ascending cholangitis

## Abstract

**Background:**

Mucinous cystic neoplasm of the liver with extrahepatic growth is a rare benign epithelial neoplasm of the biliary system that presents with a mass effect or is incidentally found on imaging. The tumor affects mostly the common hepatic duct, which is difficult to diagnose preoperatively by radiology, endoscopy, or cystic fluid analysis. Endoscopic ultrasound is a noninvasive tool for the evaluation of features of a cystic lesion and the extent of disease. Optimal treatment is complete tumor resection.

**Case presentation:**

A 27-year-old Thai woman was referred to our hospital for investigation and treatment of clinical symptoms of obstructive jaundice and ascending cholangitis, as well as an unknown cause of obstruction. Multiple investigations were performed, including endoscopic retrograde cholangiography and magnetic resonance imaging. Endoscopic ultrasound showed a multiloculated cystic lesion with internal septations without communication to the bile duct, which helped to support a diagnosis of mucinous cystic neoplasm. Eventually, the pathological diagnosis made was mucinous cystic neoplasm of the bile duct. A follow-up clinical examination with imaging at 6 months revealed that the patient was asymptomatic and without recurrence.

**Conclusions:**

We report a rare case of a patient with a large mucinous cystic neoplasm of the liver with extrahepatic growth causing biliary obstruction, which was diagnosed on the basis of endoscopic ultrasound features. Following definitive diagnosis, treatment with complete surgical resection using a multidisciplinary approach was successful.

## Background

Mucinous cystic neoplasm of the liver (MCN-L) with extrahepatic growth is a rare benign epithelial neoplasm of the biliary system. This tumor commonly originates in the intrahepatic bile duct, with less than 10% of cases occurring in the extrahepatic bile duct or gallbladder [1–3]. These tumors are found mostly in middle-aged women. The tumor usually presents with a mass effect, such as obstructive jaundice, abdominal pain, and/or ascending cholangitis. MCN-L is often benign but with malignant potential, and it is difficult to differentiate benign from malignant cases by radiology, endoscopy, or cystic fluid analysis [4, 5]. Optimal management of this cystic tumor is complete surgical resection [3, 6].

We report what is to the best of our knowledge the first published case of MCN-L with extrahepatic growth in Thailand. Our patient presented with ascending cholangitis at a local hospital and was then referred to our university teaching tertiary care hospital. We needed to perform multiple procedures for investigation and biliary drainage before arriving at a definitive diagnosis and treatment. A literature review on investigations and management of MCN-L is included.

## Case presentation

On 1 November 2016, a 27-year-old Thai woman was referred to our hospital from a local hospital with progressive jaundice, abdominal pain, and intermittent fever with chills. She had initially been treated with antibiotics, and she had seemed to recover for a few days, but the symptoms returned, and she was referred to our hospital. She was unemployed and had no history of comorbid disease, blood transfusions, intravenous drug use, or family history of viral hepatitis or liver disease. On physical examination, she appeared unwell with a high-grade fever and moderate icteric sclera. Her abdomen was soft with significant abdominal distention and mild tenderness at the epigastrium with a negative Murphy’s sign; liver 10 cm with a firm consistency, smooth surface, and blunt edge; was shifting dullness-negative; and had no cutaneous signs of chronic liver stigmata.

**Fig. 1:**
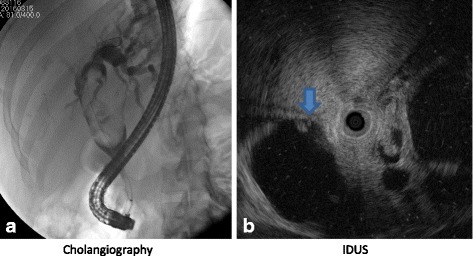
**a** Cholangiogram shows a large oval-shaped filling defect (*arrow*) in the common hepatic duct with intrahepatic duct dilation. **b** Intraductal ultrasound shows hypoechoic content in a large oval filling defect with internal septation

Results of the patient’s liver function tests were abnormal: total bilirubin 8.11 mg/dL (normal range 0.2–1.2 mg/dL), direct bilirubin 7.06 mg/dL (normal range 0–0.2 mg/dL), aspartate aminotransferase 169 U/L (normal range 0–32 U/L), alanine aminotransferase 182 U/L (normal range 0–33 U/L), and alkaline phosphatase 992 U/L (normal range 39–117 U/L). The patient’s viral hepatitis B and C profiles were negative. Magnetic resonance imaging (MRI) with magnetic resonance cholangiopancreatography (MRCP) of her abdomen had been performed at a local hospital before her referral. These images showed dilation of the left intrahepatic and common hepatic ducts with the presence of an enhancing oval-shaped filling defect 3 × 5 cm in size at the common hepatic duct, which was initially suspected to be a common hepatic duct stone. She was referred to our hospital, where she underwent endoscopic retrograde cholangiopancreatography (ERCP) for removal of the stone, which was found to be a large oval-shaped filling defect in the common hepatic duct with left intrahepatic duct dilation. The stone could not be removed. A biliary double-pigtail plastic stent was successfully placed above the filling defect for biliary drainage. A second ERCP was performed for stone removal, but the lesion was fixed and could not be removed. Intraductal ultrasound was used to identify the lesion after removal failure. These images showed hypoechoic content in a large filling defect with internal septation (Fig. 1a and b). Intraductal brushing and biopsy were done, and the results were negative for malignancy.

**Fig. 2:**
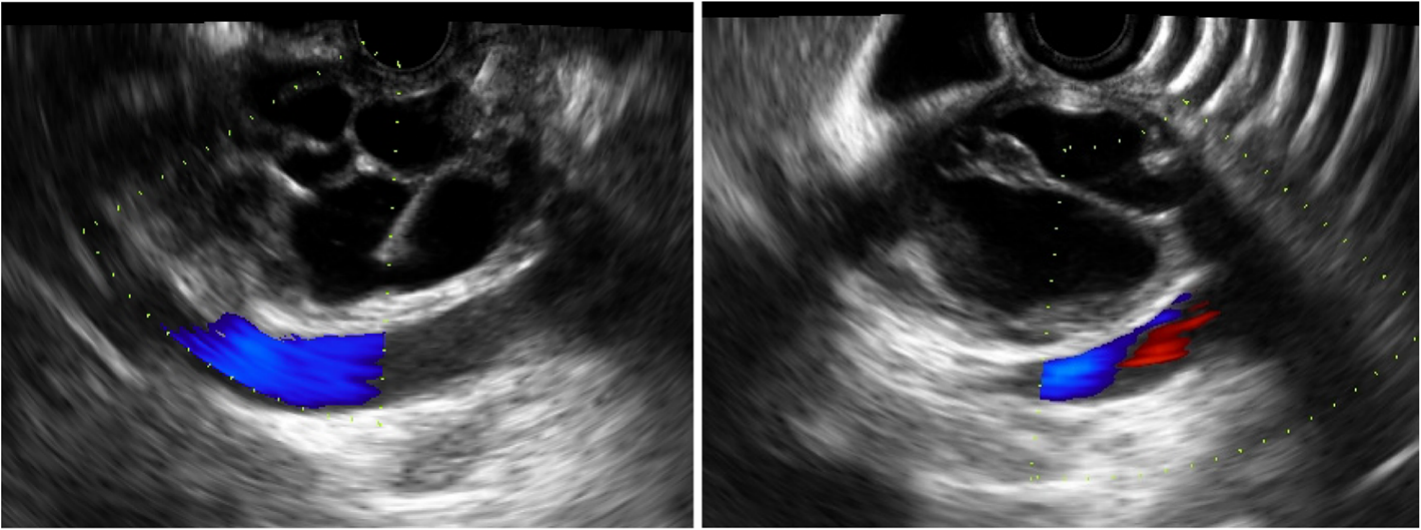
Endoscopic ultrasound shows a thin-walled, multiseptated cystic lesion in the common hepatic duct and anechoic content intracystic fluid without mural nodules or papillary projections

The advanced endoscopy team thought that this lesion might be a cystic tumor, so endoscopic ultrasound (EUS) was performed, which demonstrated dilation of the intrahepatic bile duct and a thin-walled, multiseptated cystic lesion in the common hepatic duct with anechoic intracystic fluid content and without mural nodules or papillary projections (Fig. 2). Cystic fluid aspiration was done, which revealed a fluid carcinoembryonic antigen (CEA) level of 26.41 ng/ml. The EUS features supported a diagnosis of cystic tumor of the bile duct.

**Fig. 3:**
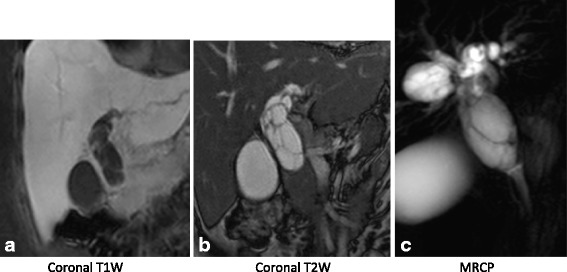
Magnetic resonance imaging (**a** = coronal T1W, **b** = coronal T2W) and magnetic resonance cholangiopancreatographic images (**c** = MRCP) show an enhancing wall, multiseptated cystic lesion about 5.6 × 2.3 cm in size in the common hepatic duct, not communicating with the common bile duct

Previous MRI with MRCP that showed an enhancing wall, multiloculated, and septated cystic lesion about 5.6 × 2.3 cm in size in the common hepatic duct, which was not communicating with the bile duct (Fig. 3a–c), was reviewed again with our abdominal radiologist.

After receiving a complete course of intravenous antibiotics and biliary drainage, the patient was clinically well with improved jaundice and fever. At her 2-week follow up, she was anorexic with abdominal distention.

**Fig. 4:**
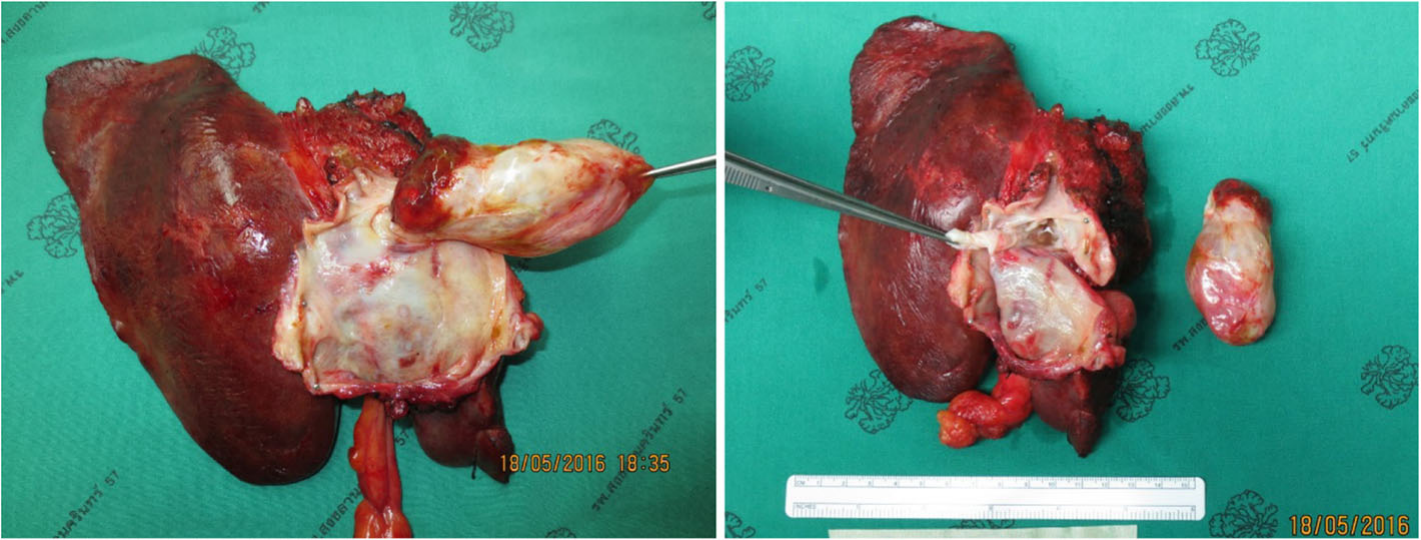
Gross examination of the surgical specimen shows an oval, thin-walled cystic tumor with internal septation in the common hepatic duct. The cystic content was a clear yellowish fluid

**Fig. 5:**
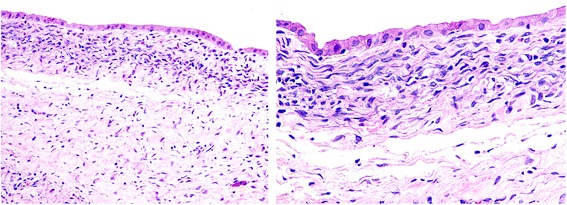
Histopathological specimens show a single layer of cuboidal epithelium resting on the basement membrane and compact, spindle-shaped cells in the mesenchymal stroma (ovarian-like stroma)

The definitive treatment was complete tumor resection and left hepatectomy. Gross examination of the surgical specimen demonstrated a large, oval, thin-walled cyst in the common hepatic duct extending to the left proximal intrahepatic duct causing biliary dilation (Fig. 4). The cystic content was clear yellowish fluid with multiseptation. Histopathology was done to confirm the diagnosis (Fig. 5).

At her 6-month follow-up after complete tumor resection, the patient was doing well, with normal liver function tests and without evidence of recurrence.

## Discussion

MCN-L is a rare benign cystic neoplasm of the biliary system, with an estimated incidence of about 5% of all hepatobiliary cystic neoplasms. This cystic tumor is very rare in the extrahepatic biliary system and gallbladder [1–3, 7]. MCN-L with extrahepatic growth can occur anywhere along the extrahepatic bile duct, most commonly in the common hepatic duct [4, 8]. These neoplasms are usually found in middle-aged women presenting with jaundice, abdominal pain, and ascending cholangitis resulting from intraluminal tumor obstruction [4, 5]. MCN-L is possibly hormone-dependent because it has been found to increase in size after beginning an oral contraceptive agent [8].

MCN-L with extrahepatic growth is usually a single, multiloculated cystic tumor characterized by the presence of a single layer of cuboidal or nonciliated columnar epithelium resting on a basement membrane (Fig. 5). Mesenchymal (ovarian-like) stroma is seen in 85% of cases, consisting of a thick layer of compact, spindle-shaped cells and supported by the epithelium [1–3, 9]. Biliary cystadenoma with ovarian-like stroma is considered benign with malignant potential but has a good prognosis, whereas the prognosis is less good for cases without ovarian-like stroma [2, 3, 10]. The histopathology is similar to that of mucinous cystic tumors in the pancreas and ovaries.

The majority of MCN-L cases do not communicate with the bile duct [5]. Radiological diagnosis of MCN-L is possible on transabdominal ultrasonography, contrast-enhanced computed tomography (CECT)/MRI abdomen or EUS. Typical ultrasound findings are globular or ovoid, well-demarcated, noncalcified, thick-walled, multiloculated, septated internal echoes, with or without papillary projections [8, 11, 12]. On CECT studies, the abdomen shows multiloculated and septated intrabiliary cystic lesions with a well-defined capsule, and wall enhancement can be seen with or without calcifications [13]. MRI findings are low signaling intensity on T1-weighted images and high signaling intensity on T2-weighted images with enhancement of septations and cyst walls with or without calcification [14]. MRCP is used in evaluating the extent of disease and bile ducts proximal to the lesion before surgical intervention [15]. Although radiologic imaging alone cannot differentiate benign from malignant MCN-L tumors, some findings, such as irregular wall enhancement, papillary projections, and/or mural nodules, increase suspicion of malignancy [16, 17].

EUS is a noninvasive tool for diagnosis of MCN-L, with few reported cases of EUS features in this tumor. EUS can reveal significant features of an MCN-L, most notably a well-defined, multilocular cystic lesion with a thick-wall containing multiple septations or papillary projections, without communication between the cystic tumor and bile duct. EUS can identify mural nodules and wall irregularities more clearly than other imaging modalities, which can help predict the risk of malignancy. EUS intervention can also be used for cystic fluid aspiration and/or target wall biopsy before surgical planning [5, 6]. A few studies have demonstrated increased levels of serum and cystic fluid tumor markers (CEA and cancer antigen 19-9), which can help to differentiate the diagnosis of MCN-L from a simple cyst, but they do not help to exclude malignancy [18]. A preoperative core needle biopsy to detect malignancy is not recommended, because this does not provide adequate information and increases the risk of needle seeding and dissemination [1, 19].

ERCP is useful for diagnosis and therapeutic procedures in biliary cystadenoma in patients who present with obstructive jaundice, ascending cholangitis, or hemobilia. The ERCP finding in MCN-L with extrahepatic growth is a well-demarcated filling defect with proximal bile duct dilation (obstruction) and a tumor that is not communicating with the bile duct [5, 8].

MCN-L with extrahepatic growth is potentially malignant, for which complete surgical resection is the treatment of choice with the lowest recurrence rate [3, 6]. The prognosis of this tumor is excellent after complete tumor resection. Local recurrence or malignant transformation is possible and often related to incomplete resection. Follow-up with radiologic imaging is necessary.

## Conclusions

We report a rare case of a large MCN-L with extrahepatic growth causing biliary obstruction that was diagnosed on the basis of EUS features. Following definitive diagnosis, we were successful with complete surgical resection using a multidisciplinary approach.

## Abbreviations

CEA: Carcinoembryonic antigen
CECT: Contrast-enhanced computed tomography
ERCP: Endoscopic retrograde cholangiopancreatography
EUS: Endoscopic ultrasound
MCN-L: Mucinous cystic neoplasm of the liver
MRCP: Magnetic resonance cholangiopancreatography
MRI: Magnetic resonance imaging
